# An Easy and Rapid Transformation Protocol for Transient Expression in Cotton Fiber

**DOI:** 10.3389/fpls.2022.837994

**Published:** 2022-03-22

**Authors:** Xiaoguang Shang, Lijie Zhu, Yujia Duan, Qingfei He, Meiyue Zhao, Yujia Yu, Wangzhen Guo

**Affiliations:** State Key Laboratory of Crop Genetics and Germplasm Enhancement, Cotton Germplasm Enhancement and Application Engineering Research Center, Ministry of Education, Nanjing Agricultural University, Nanjing, China

**Keywords:** cotton fiber, *Agrobacterium*, transient transformation, promoter activity, subcellular localization

## Abstract

Cotton fiber is the most important natural textile material in the world. Identification and functional characterization of genes regulating fiber development are fundamental for improving fiber quality and yield. However, stable cotton transformation is time-consuming, low in efficiency, and technically complex. Moreover, heterologous systems, such as *Arabidopsis* and tobacco, did not always work to elucidate the function of cotton fiber specifically expressed genes or their promoters. For these reasons, constructing a rapid transformation system using cotton fibers is necessary to study fiber’s specifically expressed genes. In this study, we developed an easy and rapid *Agrobacterium*-mediated method for the transient transformation of genes and promoters in cotton fibers. First, we found that exogenous genes could be expressed in cotton fibers *via* using β-glucuronidase (GUS) and green fluorescence protein (GFP) as reporters. Second, parameters affecting transformation efficiency, including LBA4404 *Agrobacterium* strain, 3 h infection time, and 2-day incubation time, were determined. Third, four different cotton genes that are specifically expressed in fibers were transiently transformed in cotton fibers, and the transcripts of these genes were detected ten to thousand times increase over the control. Fourth, GUS staining and activity analysis demonstrated that the activity profiles of *GhMYB212* and *GhFSN1* promoters in transformed fibers are similar to their native activity in developmental fibers. Furthermore, the transient transformation method was confirmed to be suitable for subcellular localization studies. In summary, the presented *Agrobacterium*-mediated transient transformation method is a fast, simple, and effective system for promoter characterization and protein expression in cotton fibers.

## Introduction

Cotton is an important industrial crop in the world. Cotton fiber, a highly elongated and thickened single cell of the seed epidermis, is the world’s most important natural textile raw material (Kohel et al., 1970). Cotton fiber development can be divided into four distinct but overlapping stages, namely, differentiation, elongation, secondary cell wall (SCW) thickening, and maturation (Graves and Stewart, 1988). On the day of anthesis, approximately one-third of the epidermal cells of the cotton ovule will differentiate into unbranched fiber cells (Stewart, 1975). During the elongation stage, cotton fiber cells elongate to ∼3 cm in 20 days before they switch to intensive secondary cell wall cellulose synthesis (Ruan, 2007). After maturation, more than 95% of fiber weight is cellulose. Due to these cellular and developmental characteristics, in addition to its important industrial value, the cotton fiber represents an outstanding single-cell model to study the control of cell differentiation, rapid elongation, and cellulose synthesis. Mining key genes, investigating their expression pattern and biological functions in fiber cell development will not only contribute to improving the yield and quality of cotton fibers but also will benefit the plant science communities.

Stable cotton transformation is the most revealing method for characterizing the gene functions and promoter activities in fiber cells. A few genes and promoters have been stably transformed into cotton to detect their functions in cotton fiber development (Deng et al., 2012; Lv et al., 2015; Zhang et al., 2018; Sun et al., 2019). However, cotton transformation is time-consuming, low in efficiency, and technically complex (Zhang, 2013). Transformation of cotton genes or promoters into model plants, such as *Arabidopsis thaliana* and tobacco, was an alternative way to investigate biological function. A previous study showed that an R2R3-MYB transcription factor that was specifically expressed in cotton fibers was transformed into *Arabidopsis* and affected the secondary cell wall biosynthesis and deposition in the transgenic plants (Sun et al., 2015). In addition, a cotton fiber-preferential promoter, p*GbEXPA2*, was verified to be regulated by gibberellins and abscisic acid *via* transformation in *Arabidopsis* (Li et al., 2015). The promoter of a cotton lipid transfer protein gene, *FSltp*4, showed strong activity in all types of trichomes in transgenic tobacco plants (Delaney et al., 2007). Despite these advances, promoters of many genes specifically expressed in cotton fibers have very low activities or are not active in these model plants, maybe due to the heterologous system of different species. Also, the functions of cotton fiber-specific genes cannot be fully demonstrated *via* ectopic expression in model plants. Establishing a simple, fast, and efficient transformation method in cotton fiber would greatly facilitate the studies on cotton fiber development.

*Agrobacterium*-mediated transient transformation, which is much more versatile, quick, and efficient in comparison with stable transformation, has been enormously applied for gene functional studies or biological material production in plants, including gene expression studies (Merle et al., 2002), promoter characterization (Yang et al., 2000; Rancé et al., 2002), gene silencing (Dubey et al., 2017), elicitor identification (Palanichelvam et al., 2000), vaccine production (D’Aoust et al., 2008), and CRISPR/Cas9-based genome editing (Kaur et al., 2021). *Agrobacterium*-mediated transient transformation is implemented *via* agroinfiltration. Generally, syringe and vacuum infiltration, which are applicable for a variety of plant species, are the mainly used methods for agroinfiltration. Genes are successfully transiently expressed in various tissues of different plants *via* syringe infiltration, such as *Arabidopsis*, soybean leaves (Kim et al., 2009; King et al., 2015), and melon fruit (Han et al., 2015). Vacuum infiltration has also been utilized for transient transformation in different plant tissues, including tobacco, black pepper leaves (Mani and Manjula, 2011; Fujiuchi et al., 2016), and lisianthus pollen (Sung, 2004). Although specialized equipment is required, vacuum infiltration exhibits improved transformation potential or yield (Zhao et al., 2017).

In this study, we developed an easy and rapid *Agrobacterium*-mediated transient transformation method in cotton fibers that can be used for promoter characterization and protein subcellular localization. By using the optimized vacuum infiltration method, we successfully overexpressed four previously reported cotton genes, analyzed the activities of two promoters, and performed the subcellular localization assay. The established method will provide an easy and fast way to perform functional studies on cotton fiber preferentially or specifically expressed genes.

## Materials and Methods

### Plant Materials

The allotetraploid cotton cultivar *Gossypium hirsutum* (accession TM-1) was used in this study. Cotton plants were cultivated in a glasshouse with a photoperiod of 14 h/10 h (light/dark) and a temperature of 28°C of day and 20°C of night. Cotton boll age was determined by tagging each pedicel on the day of flowering. Cotton bolls were collected on selected days post-anthesis (DPA). Fresh cotton fibers attached to seeds were used for *Agrobacterium* infiltration.

### Plasmid Construction

To test whether genes can be expressed in cotton fibers *via Agrobacterium*-mediated transient transformation, binary overexpressing vectors were constructed. Coding sequences (CDS) of *GhMYB212* (Gh_D11G3078) (Sun et al., 2019), *GhCFE1* (Gh_A05G1404) (Lv et al., 2015), *GhSusC* (Gh_D06G0825) (Brill et al., 2011), and *GhFSN1* (Gh_A12G1049) (Zhang et al., 2018) were cloned from *G. hirsutum* TM-1 and inserted downstream of the constitutive CaMV35S promoter in pBI121 vector, respectively. Additionally, the 1,944 bp promoter sequences of *GhMYB212* and 2,035 bp promoter sequences of *GhFSN1* were replaced by the CaMV35S promoter in the pBI121 vector, respectively, to construct the recombinant plasmids of p*GhMYB212*::GUS and p*GhFSN1*::GUS.

All the plasmids were introduced into the *Agrobacterium* cells as described previously (Chen et al., 1994). The primers used for vector construction are listed in Supplementary Table 1.

### *Agrobacterium*-Mediated Transient Transformation of Cotton Fibers

Transformed *Agrobacterium* was cultured at 28°C in liquid Luria-Bertani (LB) medium (supplemented with 50 μg/mL rifampicin and 50 μg/mL kanamycin) until the OD_600_ reached 1.5. Then, the *Agrobacterium* cells were collected by centrifugation (4,000 rpm, 10 min) and resuspended with transformation solution [1/2 Murashige and Skoog (MS) medium supplemented with 165 μM acetosyringone and 3% sucrose (w/v), pH = 5.8] to an OD_600_ between 0.8 and 0.9. Before the resuspension procedure, the transformation solution was degassed with a vacuum pump (Sanplatec Inc., Satoshi Kato, Japan) under 0.07 MPa for 3 min.

**FIGURE 1:**
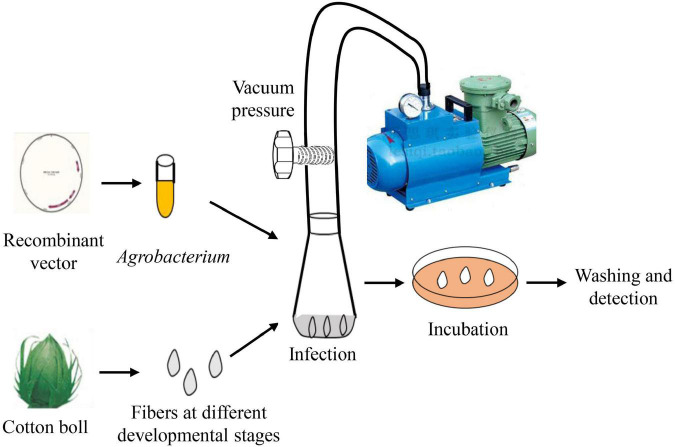
Schematic procedures for *Agrobacterium*-mediated transient transformation of cotton fibers.

Cotton bolls at selected DPA were collected, sterilized with 70% ethanol, and carefully stripped off to avoid damaging to the fibers. Then, fibers attached to the seeds were placed into a conical flask containing the resuspended *Agrobacterium* solution. After being treated with a vacuum pump at a pressure of 0.07 MPa for 2 min, 0.01% (v/v) Tween 20 was added into the transformation solution. The cotton fibers were infected by *Agrobacterium* cells under dark (25°C and 50 rpm/min) conditions for a determined period of time. Later, cotton fibers were taken out and rinsed with sterile water more than five times until the water is clear. Then, the fibers were transferred to 1/2 MS medium [supplemented with 0.3% (w/v) plant gel, 50 μg/mL rifampicin, and 50 μg/mL kanamycin, pH = 5.8] and placed in an incubator with a temperature of 26°C/20°C (day/night) and a photoperiod of 16 h of light and 8 h of dark. After co-incubation, fibers were used for gene expression analysis, enzyme activity determination, and fluorescence signal observation. Figure 1 summarizes the procedure to generate transient transgenic cotton fibers.

### β-Glucuronidase Activity Detection

Cotton fibers transformed with *Agrobacterium* containing p35S::GUS, p*GhMYB212*::GUS, or p*GhFSN1*::GUS constructs were subjected to GUS histochemical staining and enzyme activity determination, with fibers transformed with empty *Agrobacterium* as the negative control. The GUS staining and activity assay were performed according to Jefferson et al. (1987). Fibers on each seed were collected and used as one biological replicate. Three biological replicates were used for each reaction.

### Green Fluorescence Protein Fluorescence Observation

Coding sequences of enhanced GFP gene (eGFP) under the control of CaMV35S promoter were transiently transformed into cotton fibers. Fluorescence of eGFP was observed using a confocal laser scanning microscope (TCS SP8, Leica, Germany) with the 488 nm excitation and 495–530 nm emission wavelengths. Fibers transformed with empty *Agrobacterium* were used as the negative control.

### RNA Isolation, Quantitative Real-Time PCR, and Expression Analysis

Total RNA was isolated from transformed fibers using the Biospin Plant Total RNA Extraction Kit (Hangzhou Bioer Technology Co., Ltd., Hangzhou, China). The cDNA was synthesized from RNA using the HiScript III RT SuperMix (+gDNA wiper) (Vazyme, Inc., China) according to the manufacturer’s instructions. Gene-specific primers for quantitative reverse transcript PCR (qRT-PCR) analysis were designed using Primer Premier 5.0 software. The qRT-PCR reaction was performed on a 7500 Fast Real-Time PCR System (Applied Biosystems, Foster City, CA, United States) using AceQ SYBR Green Master (Vazyme, Inc., China). The cotton ubiquitin 7 (*GhUBQ7*, accession number: DQ116441) was used as a reference for normalization (Li et al., 2018). Relative expression levels were calculated according to the method described by Livak and Schmittgen (2001). Three biological replicates were used for each sample. Detailed primer information is shown in Supplementary Table 1.

### Subcellular Localization of Proteins in Fiber Cells

To test the application of subcellular localization in cotton fiber cells *via* the developed *Agrobacterium*-mediated transient transformation, a nuclear-localized protein histone B2, AtHTB2 (Zhang et al., 2020), and a plasma membrane-localized aquaporin, AtPIP2A (Qiu et al., 2020), were selected. Open reading frames of these two proteins without stop codon were fused with mCherry to generate 35S::*AtH2B*-mCherry and 35S::*AtPIP2A*-mCherry constructs, respectively. The recombinant vectors were transiently expressed in fiber cells by using the *Agrobacterium*-mediated method as described above. Subcellular localization was observed using a confocal laser scanning microscope at the 587 nm excitation and 610 nm emission wavelengths (TCS SP8, Leica, Germany).

## Results

### Construction of the Transient Expression Method in Cotton Fiber Cells

**FIGURE 2:**
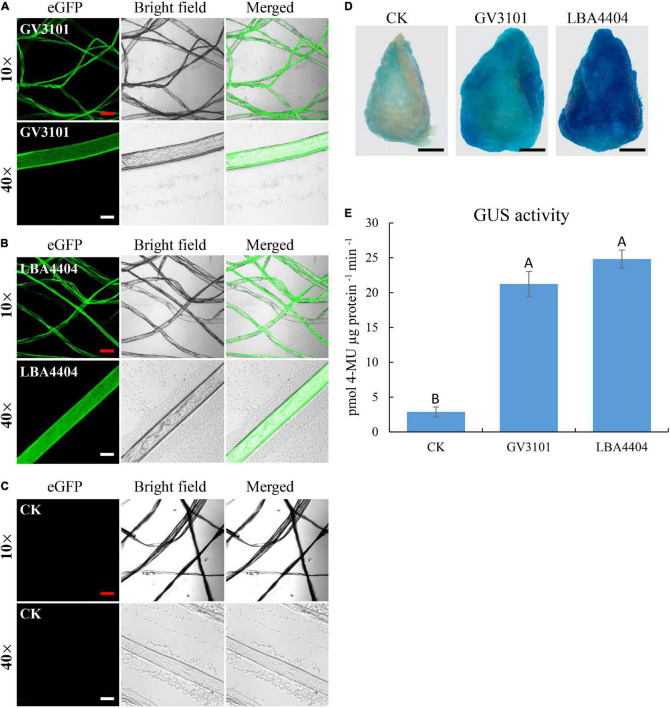
Transient expression of enhanced green fluorescence protein (GFP) gene (eGFP) and β-glucuronidase (GUS) protein in cotton fibers incubated with different *Agrobacterium* strains. **(A,B)** eGFP signal observation in cotton fibers transiently expressing 35S::eGFP construct using GV3101 **(A)** or LBA4404 **(B)**. **(C)** Fibers without *Agrobacterium* infection were used as control. The red line and white line indicate 100 and 25 μm, respectively. **(D,E)** GUS staining **(D)** and activity determination **(E)** of cotton fibers transiently expressing 35S::GUS construct using GV3101, LBA4404, or fibers without *Agrobacterium* infection. Fibers on one seed were used as one biological replicate, and at least, three independent biological replicates were detected. The black line indicates 2 mm. Different upper case letters in **(E)** indicate significant differences at *P* < 0.01 (Turkey’s test). Error bars indicate the SD of three biological replicates. Fibers at 20-day post-anthesis (DPA) were used for transformation.

As shown in Figure 1, procedures of transient expression of the protein in cotton fiber cells are involved in the screening of the target gene, *Agrobacterium* strain, fiber developmental stage, infection time, and co-incubation time. In the beginning, we selected eGFP as the target gene due to its ready observation. The plasmid containing 35S::eGFP was introduced into two commonly used *Agrobacterium tumefaciens* strains GV3101 and LBA4404, respectively. The cotton fibers at 20 DPA were selected to perform *Agrobacterium* infection. Fiber cells were infected with *Agrobacterium* for 3 h, followed by 3-day incubation. Strong eGFP fluorescence signals were observed in cotton fiber cells that were infected with GV3101 or LBA4404 (Figures 2A,B), indicating that the eGFP protein was successfully expressed in fiber cells. However, no fluorescence signals were observed in the control fibers, which were not infected with *Agrobacterium* (Figure 2C). At 10× magnification, the eGFP signals were similar between fiber cells infected with GV3101 and LBA4404; however, at 40× magnification, strong fluorescence signals were observed in the cytoplasm of fibers infected with LBA4404, while many weak signals in fibers with GV3101 (Figures 2A,B). Then, we used the GUS report system to further verify the transient expression of proteins in cotton fiber cells. GUS staining showed that the control fibers that were not infected with *Agrobacterium* exhibited weak signals (Figure 2D), which is consistent with previous reports that the enzyme β-glucuronidase is present in developing cotton fibers (Sudan et al., 2006). Further analysis demonstrated that fiber cells infected with LBA4404 or GV3101 harbors 35S::GUS construct showed much stronger GUS expression than that in the control fibers (Figure 2D). Additionally, LBA4404-inoculated fiber cells exhibited deeper staining than that in GV3101-inoculated fiber cells. GUS activity showed that fibers transformed with LBA4404 or GV3101 had higher GUS activity than the control (Figure 2D). Furthermore, LBA4404-inoculated fiber cells showed higher GUS activities than those in GV3101-inoculated fiber cells. These results suggest that the target gene could be transiently expressed in cotton fiber cells *via* the *Agrobacterium*-mediated transient transformation method, and the LBA4404 strain is more efficient to perform the fiber cell infection process. Thus, the LBA4404 strain was used in the following experiments.

**FIGURE 3:**
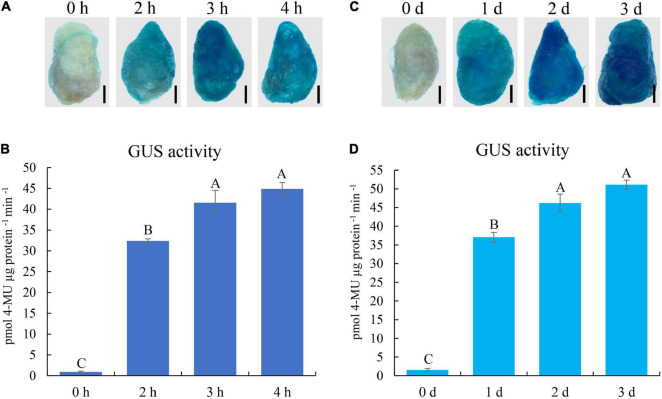
GUS expression analyses of 35S::GUS construct in cotton fibers under different transformation conditions. **(A)** GUS staining of fibers under different infection times with *Agrobacterium*. **(B)** GUS activity of cotton fibers infected with *Agrobacterium* containing 35S::GUS construct under different infection times. **(C)** GUS staining of fibers under different incubation times after *Agrobacterium* infection. **(D)** GUS activity of cotton fibers under different incubation times following *Agrobacterium* infection. The black line indicates 2 mm in **(A,C)**. Error bars indicate SD. Different letters indicate significant differences (Turkey’s test; *P* < 0.01). h, hour; d, day. Fibers at 20 DPA were used for transformation.

To determine the optimal parameters for transient expression, the infection time was investigated using the GUS report system. Cotton fibers were infected with *Agrobacterium* carrying the 35S::GUS plasmid for 0, 2, 3, and 4 h, respectively. After incubation for 1 day, GUS staining was performed on these fibers. Compared with the weak GUS staining signal in cotton fibers without *Agrobacterium* infection (0 h), 2, 3, and 4 h infection resulted in a strong GUS signal in fiber cells (Figure 3A). GUS activity further showed that the activities in 3 and 4 h infected fibers were significantly higher than that in 2 h fibers, and there was no significant difference between 3 and 4 h infected fibers (Figure 3B). Therefore, we chose 3 h infection as the optimal parameter for our transient transformation system.

We also optimized the incubation time using the GUS report system. Cotton fibers infected with *Agrobacterium* for 3 h were harvested immediately (0 day) or were further incubated on medium for 1, 2, or 3 days. GUS staining and activity determination showed that the GUS enzyme activities in cotton fibers without incubation were very weak, whereas they were much higher in 1-, 2-, or 3-day incubated fibers (Figures 3C,D). GUS activities in 2 and 3-day incubated fibers were significantly higher than that in 1-d fibers, and there was no significant difference between 2 and 3 days incubated fibers (Figure 3D). Thus, 2-day incubation was selected in our transient transformation system.

Taken together, three parameters, including LBA4404 *Agrobacterium* strain, 3 h infection time, and 2 days incubation time, were determined to study the transient expression in cotton fibers.

### Transient Overexpression of Four Cotton Fiber Preferentially Expressed Genes *via Agrobacterium*-Mediated Method

**FIGURE 4:**
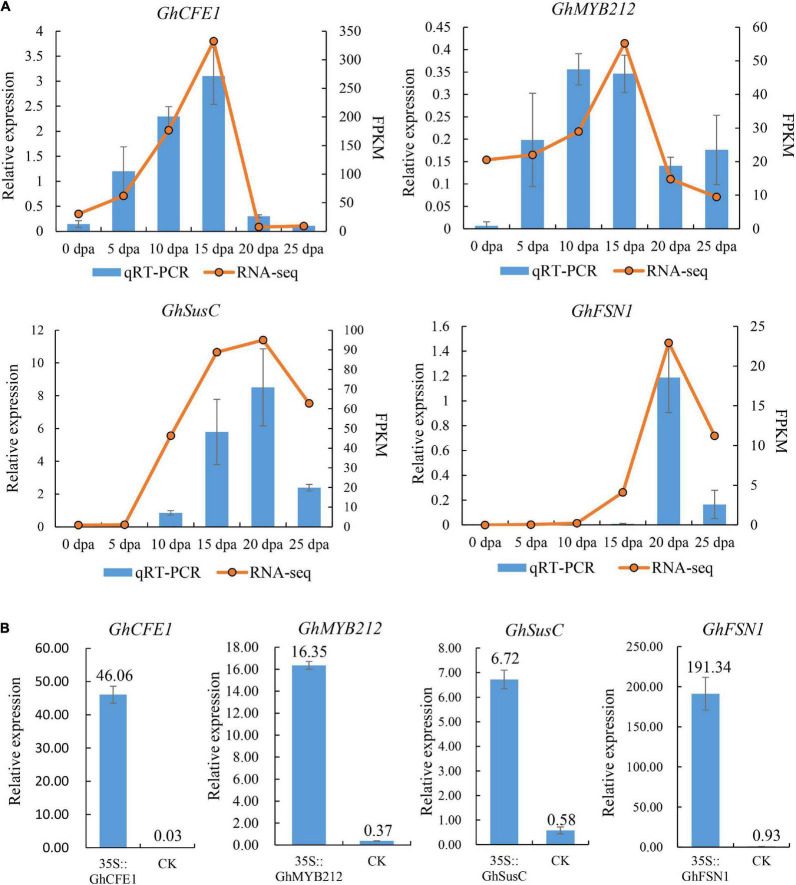
Transient overexpression of four cotton genes in fibers. **(A)** The expression pattern of four genes used for transient overexpression experiment was investigated in *Gossypium hirsutum* TM-1, including two genes, *GhCFE* and *GhMYB212*, which are preferentially expressed in elongating fibers, and two genes, *GhSusC* and *GhFSN1*, which are preferentially expressed in secondary cell wall depositing fibers. **(B)** The expression levels of *GhCFE1*, *GhMYB212*, *GhSusC*, and *GhFSN1* were significantly higher in transiently transformed fibers than that in the control, which was transformed with empty pBI121 binary expression vector, respectively. Data above each column indicate the relative expression of each gene compared with the internal reference gene, *GhUBQ7*. Fibers at 20 DPA were used for transformation. Error bars indicate SD.

We selected four previously characterized cotton genes that were preferentially expressed in fibers, including *GhMYB212* (Sun et al., 2019), *GhCFE1* (Lv et al., 2015), *GhSusC* (Brill et al., 2011), and *GhFSN1* (Zhang et al., 2018) to study the expression levels by using the transient transformation method. Expression analyses in *G. hirsutum* TM-1 showed that *GhCFE1* and *GhMYB212* were preferentially expressed in fibers undergoing rapid elongation (5–15 DPA), while *GhSusC* and *GhFSN1* were mainly expressed in fibers at the secondary cell wall thickening stage (15–25 DPA) (Figure 4A), similar to previous reports. The coding regions of these four genes were cloned and inserted downstream of the 35S promoter in the pBI121 binary expression vector to obtain 35S::*GhCFE1*, 35S::*GhMYB212*, 35S::*GhSusC*, and 35S::*GhFSN1* constructs, respectively. Furthermore, the four constructs were individually transformed into *Agrobacterium* and used for subsequent transient transformation. Fibers at 20 DPA were subjected to transformation and were collected for gene expression analysis. Quantitative RT-PCR assay showed that the transcript levels of *GhMYB212*, *GhCFE1*, *GhSusC*, and *GhFSN1* increased by 44.19, 1,535.33, 11.59, and 205.74 times in transiently transformed fibers in comparison to the control that transformed with empty pBI121 binary expression vector, respectively (Figure 4B). These results confirmed the success of our method in transient overexpressing target genes in cotton fibers.

### Promoter Activity Analysis in Fiber Cells Using *Agrobacterium*-Mediated Transient Transformation

**FIGURE 5:**
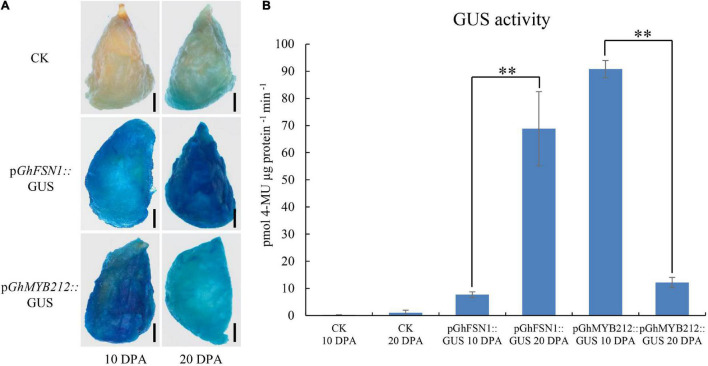
Activities of promoters of *GhFSN1* and *GhMYB212* at different fiber development stages were analyzed *via Agrobacterium*-mediated transient transformation. **(A)** GUS staining results showed that the *GhFSN1* promoter had high activity at 20 DPA fibers, while the *GhMYB212* promoter had high activity at 10 DPA fibers. Black line indicates 2 mm. **(B)** GUS activity analysis of cotton fibers infected with *Agrobacterium* containing p*GhFSN1:*:GUS or p*GhMYB212:*:GUS constructs, respectively. Error bars indicate SD. ** indicates a significant difference using the *t*-test with a *P*-value of 0.01.

To further test the efficiency of the method, we cloned the promoter regions (∼2 kb) of *GhFSN1* and *GhMYB212* into pBI121 vector upstream of GUS to make the recombinant plasmids, p*GhFSN1*::*GUS* and p*GhMYB212*::*GUS*, respectively. Cotton fibers at 10 and 20 DPA were transiently transformed with *Agrobacterium* and then were subjected to GUS staining and activity determination. The staining results revealed that weak signals were observed both in 10 and 20 DPA fibers without *Agrobacterium* infection (Figure 5A). The signal of p*GhFSN1*::*GUS* was higher in 20 DPA fibers than that in 10 DPA fibers; on the contrary, the signal of p*GhMYB212*::*GUS* was higher in 10 DPA fibers than that in 20 DPA fibers. GUS activity assays confirmed that the control fibers had low β-glucuronidase activities (Figure 5B). Again, GUS activities in 20 DPA fibers were significantly higher than that in 10 DPA fibers transformed with p*GhFSN1*::*GUS*, while GUS activities in 10 DPA fibers were significantly higher than that in 20 DPA fibers transformed with p*GhMYB212*::*GUS*. These results are consistent with the *GhFSN1* and *GhMYB212* endogenous transcript levels quantified by qRT-PCR in cotton fibers (Figure 4A), indicating that the *Agrobacterium*-mediated transient transformation method was suitable for promoter characterization in fiber cells.

### The Developed Transient Expression Method in Cotton Fiber Can Be Used for Subcellular Localization Assay

**FIGURE 6:**
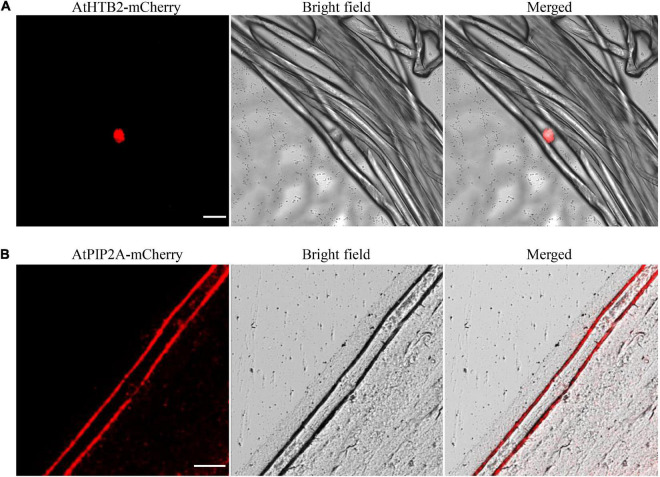
Subcellular localization of mCherry-tagged proteins in fiber cells. **(A)** A strong fluorescence signal was observed in the nucleus of fiber cells transformed with *Agrobacterium* harboring 35S::*AtHTB2*-mCherry construct. **(B)** A strong fluorescence signal was observed in the plasma membrane of fiber cells transformed with *Agrobacterium* harboring 35S::*AtPIP2A*-mCherry construct. White lines indicate 50 μm. *AtHTB2, Arabidopsis thaliana* histone B2; *AtPIP2A*, an aquaporin, short for *Arabidopsis thaliana* plasma membrane intrinsic protein. Fibers at 20 DPA were used for transformation.

Having validated that proteins could be transiently expressed in cotton fibers, we further investigated whether the expressed proteins were correctly subcellular localized in fiber cells. A nuclear-localized protein, histone B2 (AtHTB2) (Zhang et al., 2020), and a plasma membrane-localized aquaporin, AtPIP2A (Qiu et al., 2020), were ligated with mCherry protein at the N-terminal, respectively, to form 35S::*AtHTB2*-mCherry and 35S::*AtPIP2A*-mCherry constructs. *Agrobacterium*-mediated transient transformation with the 35S::*AtHTB2*-mCherry construct produced a strong fluorescence signal confined to nuclear (Figure 6A), while fiber cell expressing AtPIP2A-mCherry showed strong fluorescence at the plasma membrane (Figure 6B). These results suggested that the transient transformation method was suitable for subcellular localization studies in cotton fiber cells.

## Discussion

In this study, we developed an *Agrobacterium*-mediated transient transformation system with cotton fiber as the receptor aiming to accelerate the functional research on cotton fiber development. As far as we know, this is the first report in which the target gene was transiently expressed in cotton fiber *via* the *Agrobacterium*-mediated method. In plant research, model plants, such as *Arabidopsis thaliana* or tobacco, are generally used as transgenic receptors for gene function investigation if the species itself is difficult to make the transgenic plant, such as cotton. However, as cotton fiber has its own unique developmental characteristics, some genes or promoters specifically function in cotton fiber are expressed at very low or no expression at all in the model plants after transformation. Moreover, although different methods have been developed in cotton transformation, it is still time-consuming, labor-intensive, costly, and technically complex to investigate functions of genes and activities of promoters *via* stable genetic transformation, which has seriously hindered the research progress on the function of genes in cotton fiber development. The rapid and technically easy method described herein is supposed to partially solve this problem. In our studies, we first showed that four exogenous proteins, including GFP, GUS, AtHTB2-mCherry, and AtPIP2A-mCherry, and four cotton endogenous proteins, including GhCFE1, GhMYB212, GhSusC, and GhFSN1, were all successfully expressed in cotton fibers *via* the developed methods within a few days (Figures 2, 4, 6). Second, the promoter activities of *GhFSN1* and *GhMYB212* determined using the transient transformation method in this study were well consistent with their promoting activities in normal developing cotton fibers (Figures 4, 5), indicating that the promoter activity can be quickly and accurately determined in cotton fiber *via* the transient transformation method in this study. Additionally, AtHTB2 protein was successfully localized at nuclear and AtPIP2A at the plasma membrane in the subcellular localization assay (Figure 6), indicating that cotton fiber could be used for subcellular localization studies of the cotton endogenous proteins. In summary, these findings implied that the method developed in this study can be used for protein expression and promoter activity investigation studies.

We also optimized the transformation system by determining the affecting parameters, including the *Agrobacterium* strain, infection time, and incubation time. Through comparison of GFP fluorescence signals, GUS staining, and GUS activity determination, we showed that *Agrobacterium* strain LBA4404 had higher transformation efficiency than GV3101 (Figure 2). Furthermore, based on comprehensive consideration of expression levels of the GUS gene and operation time, infection time of 3 h, and incubation time of 2 days were suggested for performing transformation in the method (Figure 3). Based on these, we successfully developed the transient transformation system in cotton fiber with the detected infiltration parameters.

As discussed above, the method reported in this study is quite applicable for promoter characterization and *in situ* subcellular localization of proteins that are preferentially or specifically expressed in cotton fibers. Hopefully, the transient transformation of cotton fiber can be used to test the regulation of transcription factors on downstream genes and interaction studies of different proteins in fiber cells. In the future study, we will further optimize the parameters to enhance the transformation efficiency and extend its application.

Each cotton fiber is a single cell that is highly elongated and thickened from the seed epidermis. To our best knowledge, this is the first report that proteins are transiently expressed using plant single cell as the receptor *via* the *Agrobacterium*-mediated method. The results in this study imply that single intact plant cells can be transformed with *Agrobacterium*, providing important clues for future plant transformation studies.

## Conclusion

In this study, we developed an easy and fast *Agrobacterium*-mediated transient transformation method using cotton fiber as the receptor. This method can be used for *in situ* promoter characterization and subcellular localization of proteins that are preferentially or specifically expressed in cotton fibers. Furthermore, the method shows a promising application in gene functional studies, such as protein–protein interaction and regulatory network detection, after further optimization.

## Data Availability Statement

All datasets presented in this study are included in the article/Supplementary Material.

## Author Contributions

WG conceived the study and revised the manuscript. XS and WG designed the experiments. XS, LZ, YD, MZ, and YY contributed to the cotton fiber infiltration, observation, and detection. QH performed the quantitative RT-PCR analysis. XS interpreted the data and wrote the manuscript. All authors contributed to the article and approved the submitted version.

## Conflict of Interest

The authors declare that the research was conducted in the absence of any commercial or financial relationships that could be construed as a potential conflict of interest.

## Publisher’s Note

All claims expressed in this article are solely those of the authors and do not necessarily represent those of their affiliated organizations, or those of the publisher, the editors and the reviewers. Any product that may be evaluated in this article, or claim that may be made by its manufacturer, is not guaranteed or endorsed by the publisher.
